# Impact of non-pharmaceutical interventions and vaccination on COVID-19 outbreaks in Nunavut, Canada: a Canadian Immunization Research Network (CIRN) study

**DOI:** 10.1186/s12889-022-13432-1

**Published:** 2022-05-25

**Authors:** Thomas N. Vilches, Elaheh Abdollahi, Lauren E. Cipriano, Margaret Haworth-Brockman, Yoav Keynan, Holden Sheffield, Joanne M. Langley, Seyed M. Moghadas

**Affiliations:** 1 Agent-Based Modelling Laboratory, York University, Toronto, ON Canada; 2 Ivey Business School and Department of Epidemiology and Biostatistics, Schulich School of Medicine and Dentistry, Western University, London, ON Canada; 3 Rady Faculty of Health Sciences, National Collaborating Centre for Infectious Diseases, University of Manitoba, Winnipeg, MB Canada; 4 Department of Medical Microbiology, Max Rady College of Medicine, University of Manitoba, Winnipeg, MB Canada; 5 Department of Paediatrics, Qikiqtani General Hospital, Iqaluit, NT Canada; 6 Canadian Center for Vaccinology, IWK Health Centre, Nova Scotia Health Authority, Dalhousie University, Halifax, NS Canada

**Keywords:** SARS-CoV-2, COVID-19, Non-pharmaceutical interventions, Vaccination, Agent-based simulations

## Abstract

**Background:**

Nunavut, the northernmost Arctic territory of Canada, experienced three community outbreaks of the coronavirus disease 2019 (COVID-19) from early November 2020 to mid-June 2021. We sought to investigate how non-pharmaceutical interventions (NPIs) and vaccination affected the course of these outbreaks.

**Methods:**

We used an agent-based model of disease transmission to simulate COVID-19 outbreaks in Nunavut. The model encapsulated demographics and household structure of the population, the effect of NPIs, and daily number of vaccine doses administered. We fitted the model to inferred, back-calculated infections from incidence data reported from October 2020 to June 2021. We then compared the fit of the scenario based on case count data with several counterfactual scenarios without the effect of NPIs, without vaccination, and with a hypothetical accelerated vaccination program whereby 98% of the vaccine supply was administered to eligible individuals.

**Results:**

We found that, without a territory-wide lockdown during the first COVID-19 outbreak in November 2020, the peak of infections would have been 4.7 times higher with a total of 5,404 (95% CrI: 5,015—5,798) infections before the start of vaccination on January 6, 2021. Without effective NPIs, we estimated a total of 4,290 (95% CrI: 3,880—4,708) infections during the second outbreak under the pace of vaccination administered in Nunavut. In a hypothetical accelerated vaccine rollout, the total infections during the second Nunavut outbreak would have been 58% lower, to 1,812 (95% CrI: 1,593—2,039) infections. Vaccination was estimated to have the largest impact during the outbreak in April 2021, averting 15,196 (95% CrI: 14,798—15,591) infections if the disease had spread through Nunavut communities. Accelerated vaccination would have further reduced the total infections to 243 (95% CrI: 222—265) even in the absence of NPIs.

**Conclusions:**

NPIs have been essential in mitigating pandemic outbreaks in this large, geographically distanced and remote territory. While vaccination has the greatest impact to prevent infection and severe outcomes, public health implementation of NPIs play an essential role in the short term before attaining high levels of immunity in the population.

**Supplementary information:**

The online version contains supplementary material available at 10.1186/s12889-022-13432-1.

## Introduction

The COVID-19 pandemic has inflicted a devastating toll on human health, as well as causing significant socio-economic upheaval globally [1, 2]. During the first wave, many countries implemented unprecedented movement restrictions and physical-distancing measures to “flatten” outbreaks and avert an immediate burden on the health care system [3–5]. In Canada, these measures markedly attenuated the potential impact of disease, and prevented the importation or spread of SARS-CoV-2 in northern populations and Indigenous communities [6]. For instance, until November 6, 2020, no cases of COVID-19 were recorded in the territory of Nunavut, a vast Canadian Arctic region with a population of approximately 39,000 that is primarily inhabited by Indigenous Inuit peoples.

Beginning in March 2020, the Government of Nunavut instituted significant public health measures (referred to here as non-pharmaceutical interventions; NPIs) [7], including mandatory two-week isolation in specific hubs (hotels outside Nunavut) for traveling territory residents prior to re-entering Nunavut. Aside from essential workers, non-residents were not permitted into the territory regardless of their ability to isolate. On March 17, 2020 daycares and schools were closed, and on April 21 the remainder of the school year was canceled. Starting in June 2020 some restrictions were gradually lifted. The first COVID-19 outbreak in Nunavut occurred in November 2020, some 9 months into the pandemic. In response, NPIs were enhanced in the affected communities by closing non-essential businesses and schools, prohibiting indoor gatherings, lockdowns in affected regions (i.e., in Qikiqtaaluk, Kivalliq or Kitikmeot, as appropriate) and then a territory-wide lockdown in mid-November. COVID-19 vaccination started on January 6, 2021 in Nunavut at which point the November outbreak had ended, and some NPIs were concomitantly lifted. Isolation hubs remained in place until mid-June 2021 when residents with two doses of COVID-19 vaccine no longer needed to isolate before re-entering the territory. A second outbreak in the territory began in January 2021 which lasted about 7 weeks. The third outbreak began mid-April in the capital city of Iqaluit [8], and lasted for 6 weeks. A local outbreak of the highly transmissible Delta variant also occurred in the Mary River Mine in April, but remained confined due to implementation of NPIs.

In Nunavut about 63% of residents were fully vaccinated (two doses) as of December 22, 2021 compared to a Canada-wide coverage rate of over 76% of eligible citizens [9]. Importantly, Nunavut’s population is the youngest in the country with 41% of Nunavummiut being between the ages of 0–19 compared to 22% for the rest of Canada. While the vaccine program for those 16 years of age and older began in Dec 2020, 12 to 15 year olds were not eligible for COVID-19 vaccines until May 2021, and 5 to 11 year olds until November 2021. Thus Nunavut’s young population distribution altered the total percent of the population that was eligible early on during vaccine distribution [10].

Currently approved vaccines against COVID-19 in Canada have proven to be highly effective and safe, dramatically reducing the number of severe illnesses, hospitalizations, and deaths [11]. Vaccine-induced immunity in adults can be seen to be even more important in Nunavut than in southern Canada given the expected low level of naturally-acquired immunity due to outbreaks, and the smaller percentage of the population able to be vaccinated early in the vaccine rollout.

We sought to evaluate the effect of NPIs and vaccination on COVID-19 outbreaks in Nunavut. For this purpose, we employed our previously established agent-based model of COVID-19 transmission [12], and parameterized it with demographics of Nunavut, encapsulating age distribution and household composition based on census data [13]. Implementing timelines of interventions and simulating disease transmission dynamics, we show the pivotal role that NPIs played in containing the November 2020 pre-vaccine COVID-19 outbreak in Nunavut. We further investigated the role of vaccination in later outbreaks of COVID-19 and potential community spread of highly transmissible variants in the territory.

## Methods

Data for this analysis was obtained from publicly available records [14], and sources are cited throughout the paper. A team member (HS) is a local leader within the territorial healthcare system and practising clinician in Iqaluit, Nunavut. Through his role embedded within the government's health system and through existing partnerships with the greater community, he will be leading the dissemination and communication of information learned in this project guided by local context. The entire research team and the partner organization of the National Collaborating Centre for Infectious Diseases are also available to assist with ongoing knowledge translation, as directed and requested by the local community.

### The model

We adapted our previous agent-based model of COVID-19 transmission [12] to include the household structure in addition to the age distribution of the Nunavut population. We considered six age groups of 0–4, 5–19, 20–49, 50–64, 65–79, and 80 + , and distributed the model population according to census data [13], with 1, 2, 3, 4, and 5 + individuals. For households with 5 + members, we set the maximum size to 15 persons per household and selected the exact household size randomly using a uniform distribution. The model population size was 39,353, with an average of 3.8 people per household. We assumed that each household includes at least one adult individual aged 20 or older. The daily number of contacts was sampled from a negative-binomial distribution derived from CONNECT (Contact and Network Estimation to Control Transmission), using Canadian mixing matrices during the pre-pandemic period and the 2020 spring lockdown [15].

**Fig. 1:**
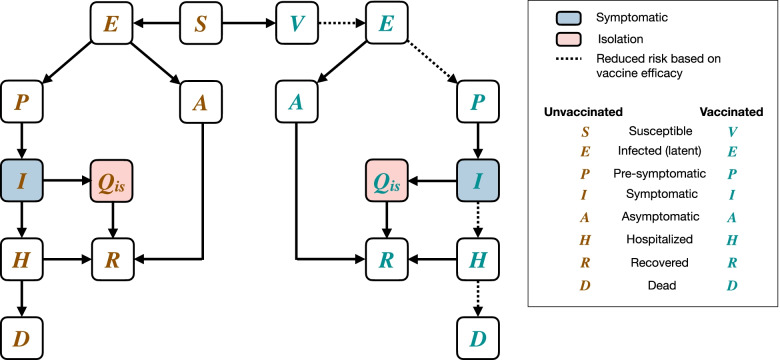
Schematic representation of the COVID-19 transmission dynamics and vaccination implemented in the model

The model encompasses the natural history of disease and classifies individuals with their epidemiological statuses as susceptible; exposed (not yet infectious); asymptomatic (and infectious); pre-symptomatic (and infectious); symptomatic with either mild or severe illness; recovered; and dead (Fig. 1). Disease transmission occurs as a result of contacts between susceptible and infectious individuals in asymptomatic, pre-symptomatic, and symptomatic stages.

### Disease dynamics and outcomes

In our model, the risk of infection for susceptible individuals depended on their contact with infectious individuals that are asymptomatic, pre-symptomatic, or symptomatic [16]. We parameterized the infectivity of asymptomatic, mild symptomatic, and severe symptomatic individuals to be 26%, 44%, and 89% relative to individuals in pre-symptomatic stage of the disease [16–18]. For each newly infected individual, the incubation period was sampled from a Lognormal distribution with a mean of 5.2 days [19]. A proportion of infected individuals progressed through the pre-symptomatic stage and ultimately developed to symptomatic. The durations of the pre-symptomatic and symptomatic stages were sampled from Gamma distributions with a mean of 2.3 days and 3.2 days, respectively [17, 20, 21]. We assumed that non-hospitalized symptomatic cases self-isolated within 24 h of symptom onset, and reduced their daily number of contacts by an average of 74%. Those who did not develop symptomatic disease remained asymptomatic until recovery, with an infectious period that was sampled from a Gamma distribution with a mean of 5 days [21, 22]. Recovery from infection was assumed to confer immunity against reinfection for the remainder of simulation timelines.

### Vaccination

Since the Moderna COVID-19 vaccine SpikeVax™ was the main vaccine used in adults in Nunavut we implemented a two-dose vaccination campaign with Spikevax™ and a rollout strategy corresponding to the daily number of vaccine doses administered in Nunavut [9]. For the study period October 27, 2021 to June 15, 2022 only Spikevax™ vaccines were distributed to Nunavut and individuals aged 18 and older (63.2% of the population) were eligible to be vaccinated. We prioritized vaccination sequentially for healthcare workers and elders, adults with comorbidities, those aged 65 and older; and individuals aged 18–64, as per local practice [23, 24]. We used a 28-day between-dose vaccine interval with efficacy estimates against infection, symptomatic disease and severe disease summarized in Table 1.

**Table 1:** Estimates of SpikeVax™ vaccine efficacy against infection, symptomatic disease, and severe disease caused by the original and Alpha variants of SARS-CoV-2 virus [25–29]

Efficacy against (%)	Infection (95% CI)		Symptomatic infection (95% CI)		Severe disease (95% CI)	
Variant	14 d after dose 1	7 d after dose 2	14 d after dose 1	7 d after dose 2	14 d after dose 1	7 d after dose 2
Original	61 (31—79)	93.3 (85.7—97.4)	92.1 (68.8—99.1)	94.1 (89.3—96.8)	92.1 (68.8—99.1)	100
Alpha	54.7 (44.8—62.9)	86 (81—90.6)	88.1 (83.7—91.5)	91 (84—95)	81.6 (71.0—88.8)	95.7 (73.4—99.9)

### Back-calculation of incidence

**Fig. 2:**
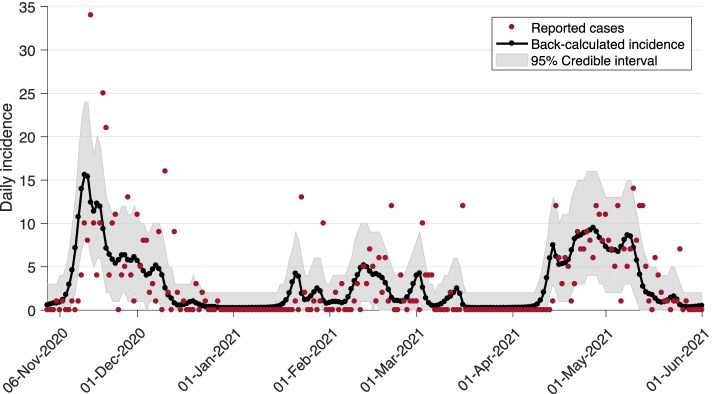
Inferred infections (black curve) from back-calculation method with 95% credible interval (shaded area) from MCMC simulations. Red dots represent the reported cases in publicly available data

We used a Bayesian non-parametric approach to back-calculate the time series of infections based on the daily reported cases of COVID-19 in Nunavut from November 6, 2020 to June 12, 2021. We then fitted the model to inferred infections. To determine the date of infection, we let ${I}_{i}$ represent the number of infections in the ${i}^{th}$ time interval, then the reported cases on day ${i}_{,}$, ${D}_{i,}$ can satisfy the convolution equation [30, 31]:

$${D}_{i}=\sum_{j=1}^{i}{I}_{j}{p}_{i-j}$$

where $${p}_{i-j}$$ is the probability that the time-interval between infection and identification is the $$i-j$$ time interval. Considering the incubation period as a proxy for this time-interval, $${p}_{i-j}$$ can be directly calculated from the incubation period distribution, given by Lognormal(shape: 1.434, scale: 0.661). Here, we made a simplifying assumption that the distribution of the incubation period did not change over time. The observed number of infections at time $$i$$ (the calendar day $$i)$$ was modelled by a nonhomogeneous Poisson process $${I}_{i}\sim Poisson({\lambda }_{i})$$, where prior distributions for the $${\lambda }_{i}$$ were given by, $${\lambda }_{i}\sim Normal({\lambda }_{i-1}, {\sigma }^{2})$$ for $$i\ge 1$$ with $${\lambda }_{i} >o$$, and $$\sigma \sim Normal (\mathrm{1,1})$$. Back calculation was implemented in a Bayesian Markov Chain Monte Carlo (MCMC) setting using Nimble, with the R statistical environment acting as the front end. MCMC simulations were run in 5 independent chains with different initial conditions for $${\lambda }_{o,}$$ each consisting of 20,000 iterations, with a burn-in period of 10,000 iterations and a thinning factor of 10. Posterior distributions were not affected by the initial conditions or prior distributions. To assess convergence, we inspected the trace plots and applied the Gelman-Rubin convergence test by computing the potential scale reduction factors (PSRF) of posterior densities. The daily inferred infections and their 95% credible intervals from this analysis are illustrated in Fig. 2.

### Model implementation and calibration

**Fig. 3:**
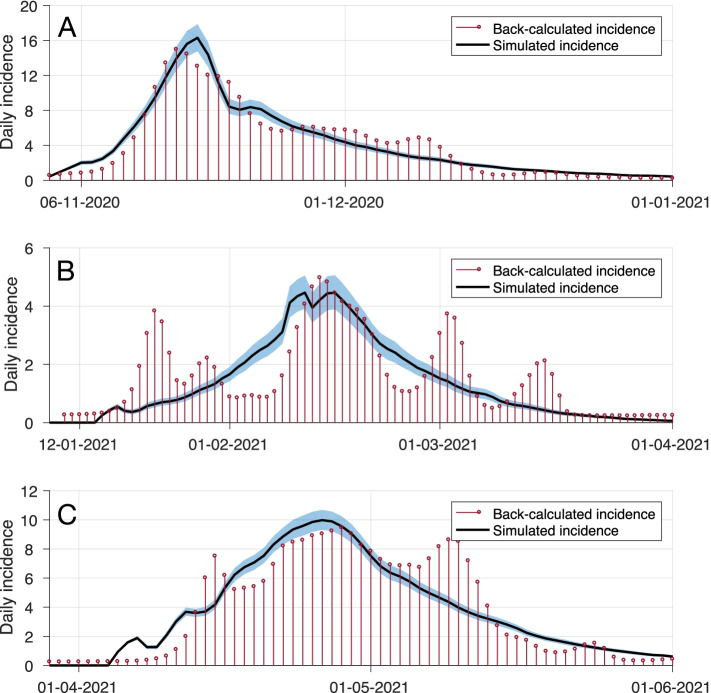
Simulated curves of the first three COVID-19 outbreaks in Nunavut, Canada, based on model fitting to the temporal cumulative incidence of infections inferred through back-calculation. The red dots represent the inferred average of infections in back-calculations from reported data. The black curve is the average of 500 Monte-Carlo simulations with 95% credible interval represented by the blue shaded area

We simulated the model with a population of 39,353 individuals, assuming no pre-existing immunity. For the first outbreak of the pandemic in Nunavut in November 2020, we used the characteristics of the original Wuhan-I strain, and determined the per-contact transmission probability (Table S1) by fitting the model to the temporal cumulative infections inferred from back-calculations during the exponential growth phase. We then adjusted the average number of daily contacts according to timelines of NPIs implemented during the outbreaks in Nunavut [32]. For the second outbreak in January 2021 (after the start of vaccination) and the Iqaluit outbreak in mid-April 2021, we considered the Alpha (B.1.1.7) variant in the model with a 50% higher transmissibility compared to the original strain simulated in the first outbreak [33, 34]. Considering the discontinued chain of transmission between these outbreaks, each outbreak was simulated separately, taking into account the population level of immunity accrued by prior infection or vaccination. We further considered a counterfactual scenario of accelerated vaccination in which 98% of Spikevax™ supply was used until 63% of the population was vaccinated. For this scenario, we considered timelines of vaccine supply delivery to Nunavut to adjust the daily rate of vaccination, accounting for a total of 2% wastage. We compared the model outcomes with the actual pace of vaccination and those obtained without vaccination. Simulation results were averaged over 500 independent Monte-Carlo realizations in each scenario, and credible intervals (CrI) were generated using the bias-corrected and accelerated bootstrap method (with 500 replications). Figure 3 shows the results of back-calculation of incidence and the model based on fitting to the temporal cumulative incidence. The model was implemented in Julia language, and simulation code is available at: https://github.com/thomasvilches/covid_nunavut

## Results

**Fig. 4:**
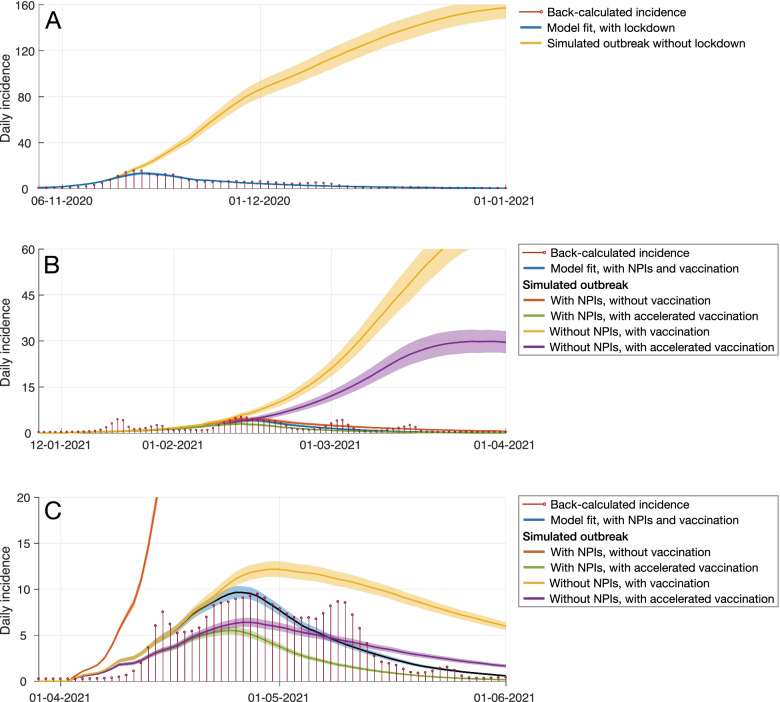
Simulated outbreaks under various scenarios in the first (**A**), second (**B**), and third (**C**) COVID-19 outbreaks in Nunavut

For the inferred infections, we observed a peak of 15.6 (95% CrI: 8.5 to 24.0) cases on November 15, 2020 during the first community outbreak in Nunavut, three days prior to the territory-wide lockdown. We found that the public health measures in Nunavut were instrumental in curbing the incidence (Fig. 4A). Without that lockdown, the model projected that Nunavut would have experienced a significantly larger outbreak with a peak incidence of 160 (95% CrI: 151—169) cases per day, which is approximately 4.7 times higher than the observed peak of 34 positive cases identified on November 17, 2020. We estimated that without a lockdown 13.5% (95% CrI: 12.5%—14.4%) of the total population would have experienced infection before the start of vaccination campaign on January 6, 2021 (Fig. 4A).

The second COVID-19 outbreak in Nunavut occurred after the start of vaccination in January 2021, with 129 reported infections. We found that with NPIs and without vaccination, the outbreak would have caused 159 (95% CrI: 133—187) infections (Fig. 4B). For the counterfactual scenario of a hypothetical accelerated vaccine roll-out, the projected outbreak size was reduced to nearly half at 77 (95% CrI: 64—100) infections. However, were NPIs not enacted, the outbreak would have led to a substantially larger proportion of the population becoming infected, 4.7% (95% CrI: 4.1%—5.2%), with projected 1,812 (95% CrI: 1,593—2,039) infections even in the presence of accelerated vaccination. We estimated that, under the pace of vaccination administered in Nunavut, without NPIs 11.0% (95% CrI: 9.9%—12.1) of the total population would have experienced infection, with a total of 4,290 (95% CrI: 3,880—4,708) infections.

We also simulated the model for the Iqaluit outbreak of the Alpha variant in mid-April (Fig. 4C) with a similar type of NPIs to the January outbreak (Fig. 4B), but with lower intensity with increasing vaccination coverage. We found that vaccination had played a pivotal role in curbing the outbreak (Fig. 4C), despite reduced efficacy against this variant. For instance, without vaccination, we projected a total of 15,196 (95% CrI: 14,798—15,591) infections would have occurred if the disease had spread through communities in Nunavut.

In the counterfactual scenario of an accelerated immunization program and with NPIs in place, the outbreak was projected to have a significantly lower number of infections at 137 (95% CrI: 123—48) cases. However, without NPIs, the outbreak size in Iqaluit would have been 1.8 times larger, causing 243 (95% CrI: 222—265) infections even in the presence of accelerated vaccination, which is comparable to the actual toll of 253 cases reported between April 15 to May 28, 2021 in Iqaluit.

## Discussion

We found that the public health measures enacted in Nunavut played a major role in preventing spread of COVID-19 in the territory. Without such measures during the first outbreak, the territorial outbreak would have had similar timelines to the second wave in southern parts of Canada, stretching over three months with a significantly higher attack rate, and a toll of severe illness. By January 31, 2021 about 5300 vaccine doses were administered in Nunavut for a coverage of 21% of the eligible population. NPIs were also critical in curbing the second outbreak of COVID-19 in January with the spread of the Alpha variant in Nunavut. Even in the scenario with a hypothetical accelerated immunization program, we found that NPIs were still required to prevent a large outbreak because of the time to generate immunity even if 11,760 vaccine doses had been administered by the end of January (i.e., ~ 47% coverage of the eligible population with the first dose). These measures were essential to interrupt the impact of the pandemic in remote communities with limited pre-existing immunity.

We found that without vaccination, the spread of the Alpha variant in the communities would have caused the largest outbreak infecting more than 38% of the population, even in the presence of NPIs. Under the vaccination pace achieved in Nunavut, we estimated that the spread of the Iqaluit outbreak through communities would have caused over 570 infections (2.2 times higher than reported cases in Iqaluit). To limit the impact of this variant further, accelerated vaccination in combination with NPIs would have been required. Clearly, NPIs remain an important public health tool, especially if there are programmatic constraints to the vaccine rollout, reduced vaccine efficacy against some SARS-CoV-2 variants, or waning immunity.

The importance of NPIs for COVID-19 outbreaks in remote and Indigenous communities has been discussed in some recent studies [35–37]. For instance, Hui et al. [37] employed an agent-based simulation model to compare NPI strategies and evaluate the effectiveness of different interventions in the setting of Indigenous communities in Australia. They found that prompt case finding, combined with quarantining of extended-household contacts and testing on exit from quarantine is the most effective strategy in remote communities with large and interconnected household composition in Australia. However, NPIs are not without harmful consequences [36]. NPIs implemented throughout the pandemic have affected employment, caregiving and support structures, mental health, and access to education [38–41]. In relation to Nunavut’s isolation hubs, as of March 2021, 10,861 individual stays took place, with a cost of 80.1 million Canadian dollars. In June, the Canadian Civil Liberties Association issued a letter to the Government of Nunavut outlining its concerns with the on-going isolation hubs [42]. The mental health effects of isolation and the resulting anxiety are identified as having health consequences for years to come. Several media reports documented hundreds of people struggling through their two-week isolation stay [43]. Internal Department of Health emails also outlined challenges in supporting vulnerable people during these stays [44].

Outbreaks of the COVID-19 pandemic continue to occur in asynchronized waves of infection worldwide with different magnitudes. In Canada, the apex of the pandemic before the emergence of the Omicron variant occurred during the third wave in spring 2021 about four months after the start of the vaccination campaign. Despite vaccine supply shortages early on, Canada has one of the highest vaccination coverage worldwide, which reduced the potential impact of the fourth wave caused by the Delta variant. Even when vaccine supply is robust, there may be suboptimal vaccine coverage in some settings. For example, by June 10, 2021, over 51,000 vaccine doses were distributed in Nunavut (enough to vaccinate all eligible residents), but only about 66% of the supply was administered [9, 45]. As mentioned previously, the high percentage of population under the age of 19 is a likely explanation for relatively lower vaccination coverage. The Comirnaty™ Pfizer-BioNTech vaccine for 12 to 15 year olds approved in Canada on May 5 were distributed to the territory as of mid-June 2021.

Our study has several limitations. First, for some model parameters, the underlying distributions were not available so were simplified to be constant based on published estimates (e.g., mean vaccine efficacies, relative transmissibility of the Alpha variant). The dynamics of disease transmission was implemented over the entire Nunavut population without consideration of a metapopulation structure with distinct communities having particular customs, interests, histories, or unique age-structured social networks. Our study does not include local perspectives of the pandemic effects, nor of consequences of the interventions on families and communities, which are critical to acceptability of pandemic mitigation strategies. Furthermore, we did not consider the location of disease transmission (e.g., within households, workplaces, or schools). For contacts outside the household, inclusion of community structure may affect our results quantitatively, but we expect the qualitative aspects of our findings to remain intact, since the model calibration and fitting to incidence data would modulate the average per-contact transmission probability. Lastly, we did not consider waning of naturally-acquired or vaccine-elicited immunity over the relatively short time horizons considered in this study.

## Conclusions

Our results demonstrate the importance of NPIs in mitigating pandemic outbreaks, indicating that measures taken by public health in Nunavut and its population reduced the impact of the pandemic according to this analysis. Vaccination remains the most effective strategy to interrupt the chain of transmission and prevent morbidity, death, and social harm. The emergence of fast-spreading Omicron SARS-CoV-2 variant, combined with waning immunity and reports of reinfections and breakthrough infections [46–48], highlighted the importance of efforts to rapidly increase vaccination coverage and enhance population immunity through booster doses in order to mitigate potential severe outcomes.

## Supplementary information

**Additional file 1:** **Table S1.** Description of model parameters and their estimates

## Data Availability

All data and computational model components are publicly available at: https://github.com/thomasvilches/covid_nunavut.

## Abbreviation

NPI: Non-pharmaceutical intervention
