# Supplementary material for: Impact of non-pharmaceutical interventions and vaccination on COVID-19 outbreaks in Nunavut, Canada: a Canadian Immunization Research Network (CIRN) study
Source: BMC Public Health. 2022 May 25;22:1042. doi: 10.1186/s12889-022-13432-1 (PMC9130454; doi:10.1186/s12889-022-13432-1)
Supplement: Supplementary file 1 — Additional file 1: Table S1. Description of model parameters and their estimates [file 12889_2022_13432_MOESM1_ESM.docx]

**Supplementary Information**

**Impact of non-pharmaceutical interventions and vaccination on COVID-19 outbreaks in Nunavut, Canada: a Canadian Immunization Research Network (CIRN) study**

**Thomas N. Vilches,^1^ Elaheh Abdollahi,^1^ Lauren E. Cipriano,^2^ Margaret Haworth-Brockman,^3^ Yoav Keynan,^4^ Holden Sheffield,^5^ Joanne M. Langley,^6^ Seyed M. Moghadas^1^**

^1^ Agent-Based Modelling Laboratory, York University, Toronto, Ontario, Canada

^2^ Ivey Business School and Department of Epidemiology and Biostatistics, Schulich School of Medicine and Dentistry, Western University, London, Ontario, Canada

^3^ National Collaborating Centre for Infectious Diseases, Rady Faculty of Health Sciences, University of Manitoba, Winnipeg, Manitoba, Canada

^4^ Department of Medical Microbiology, Max Rady College of Medicine, University of Manitoba, Winnipeg, Manitoba, Canada

^5^ Department of Paediatrics, Qikiqtani General Hospital, Iqaluit, Nunavut, Canada

^6^ Canadian Center for Vaccinology, IWK Health Centre and Nova Scotia Health Authority, Dalhousie University, Halifax, Nova Scotia, Canada

**Table S1.** Description of model parameters and their estimates.

| Description | Age groups in the model | | | | | | Source |
| --- | --- | --- | --- | --- | --- | --- | --- |
|  | 0–4 | 5–19 | 20–49 | 50–64 | 65–79 | 80+ |  |
| Transmission probability per contact during presymptomatic stage | 0.075 | | | | | | Calibrated to reproduce the cases incidence |
| Incubation period (days) | LogNormal(shape: 1.434, scale: 0.661) | | | | | | [1] |
| Asymptomatic period (days) | Gamma(shape: 5, scale: 1) | | | | | | Derived from  [2, 3] |
| Presymptomatic period (days) | Gamma(shape: 1.058, scale: 2.174) | | | | | | Derived from  [4, 5] |
| Infectious period from onset of symptoms (days) | Gamma(shape: 2.768, scale: 1.1563) | | | | | | Derived from  [2, 3] |
| Proportion of infections that are asymptomatic | 0.30 | 0.38 | 0.33 | 0.33 | 0.19 | 0.19 | [6–9] |
| Proportion of symptomatic cases that exhibit mild symptoms | 0.95 | 0.90 | 0.85 | 0.60 | 0.20 | 0.20 | [10, 11] |

**References**

1. Li Q, Guan X, Wu P, Wang X, Zhou L, Tong Y, et al. Early transmission dynamics in Wuhan, China, of novel coronavirus–infected pneumonia. N Engl J Med. 2020.
2. Li R, Pei S, Chen B, Song Y, Zhang T, Yang W, et al. Substantial undocumented infection facilitates the rapid dissemination of novel coronavirus (SARS-CoV-2). Science. 2020;368:489–93.
3. Gatto M, Bertuzzo E, Mari L, Miccoli S, Carraro L, Casagrandi R, et al. Spread and dynamics of the COVID-19 epidemic in Italy: Effects of emergency containment measures. Proc Natl Acad Sci. 2020;117:10484–91.
4. Moghadas SM, Fitzpatrick MC, Sah P, Pandey A, Shoukat A, Singer BH, et al. The implications of silent transmission for the control of COVID-19 outbreaks. Proc Natl Acad Sci. 2020;117:17513–5.
5. He X, Lau EH, Wu P, Deng X, Wang J, Hao X, et al. Temporal dynamics in viral shedding and transmissibility of COVID-19. Nat Med. 2020;26:672–5.
6. Buitrago-Garcia D, Egli-Gany D, Counotte MJ, Hossmann S, Imeri H, Ipekci AM, et al. Occurrence and transmission potential of asymptomatic and presymptomatic SARS-CoV-2 infections: A living systematic review and meta-analysis. PLoS Med. 2020;17: e1003346.
7. Mizumoto K, Kagaya K, Zarebski A, Chowell G. Estimating the asymptomatic proportion of coronavirus disease 2019 (COVID-19) cases on board the Diamond Princess cruise ship, Yokohama, Japan, 2020. Eurosurveillance. 2020;25:2000180.
8. Nishiura H, Kobayashi T, Miyama T, Suzuki A, Jung S, Hayashi K, et al. Estimation of the asymptomatic ratio of novel coronavirus infections (COVID-19). Int J Infect Dis. 2020;94:154.
9. Kimball A, Hatfield KM, Arons M, James A, Taylor J, Spicer K, et al. Asymptomatic and presymptomatic SARS-CoV-2 infections in residents of a long-term care skilled nursing facility—King County, Washington, March 2020. Morb Mortal Wkly Rep. 2020;69:377.
10. Shoukat A, Wells CR, Langley JM, Singer BH, Galvani AP, Moghadas SM. Projecting demand for critical care beds during COVID-19 outbreaks in Canada. CMAJ. 2020;192:E489–96.
11. Moghadas SM, Shoukat A, Fitzpatrick MC, Wells CR, Sah P, Pandey A, et al. Projecting hospital utilization during the COVID-19 outbreaks in the United States. Proc Natl Acad Sci. 2020;117:9122–6.
